# Plasma membrane localization of MLC1 regulates cellular morphology and motility

**DOI:** 10.1186/s13041-019-0540-6

**Published:** 2019-12-30

**Authors:** Junmo Hwang, Hung M. Vu, Min-Sik Kim, Hyun-Ho Lim

**Affiliations:** 1 Molecular Physiology and Biophysics Laboratory, Neurovascular Unit Research Group, Korea Brain Research Institute (KBRI), 41062 Daegu, Republic of Korea; 2 Department of New Biology, Daegu Gyeongbuk Institute of Science & Technology (DGIST), 42988 Daegu, Republic of Korea; 3 Department of Brain & Cognitive Sciences, Daegu Gyeongbuk Institute of Science & Technology (DGIST), 42988 Daegu, Republic of Korea

**Keywords:** MLC1, Leukodystrophy, Actin remodeling, Filopodia, Lamellipodia, Cell motility

## Abstract

**Background:**

Megalencephalic leukoencephalopathy with subcortical cysts (MLC) is a rare form of infantile-onset leukodystrophy. The disorder is caused primarily by mutations of *MLC1* that leads to a series of phenotypic outcomes including vacuolation of myelin and astrocytes, subcortical cysts, brain edema, and macrocephaly. Recent studies have indicated that functional interactions among MLC1, GlialCAM, and ClC-2 channels play key roles in the regulation of neuronal, glial and vascular homeostasis. However, the physiological role of MLC1 in cellular homeostatic communication remains poorly understood. In the present study, we investigated the cellular function of MLC1 and its effects on cell–cell interactions.

**Methods:**

MLC1-dependent cellular morphology and motility were analyzed by using confocal and live cell imaging technique. Biochemical approaches such as immunoblotting, co-immunoprecipitation, and surface biotinylation were conducted to support data.

**Results:**

We found that the altered MLC1 expression and localization led to a great alteration in cellular morphology and motility through actin remodeling. MLC1 overexpression induced filopodia formation and suppressed motility. And, MLC1 proteins expressed in patient-derived *MLC1* mutants resulted in trapping in the ER although no changes in morphology or motility were observed. Interestingly knockdown of *Mlc1* induced Arp3-Cortactin interaction, lamellipodia formation, and increased the membrane ruffling of the astrocytes. These data indicate that subcellular localization of expressed MLC1 at the plasma membrane is critical for changes in actin dynamics through ARP2/3 complex. Thus, our results suggest that misallocation of pathogenic mutant MLC1 may disturbs the stable cell-cell communication and the homeostatic regulation of astrocytes in patients with MLC.

## Introduction

Patients with MLC exhibit abnormalities in brain ion and water homeostasis, resulting in diffuse cerebral white matter signaling on spectroscopic MRI, abnormal swelling of the white matter, and macrocephaly [1–3]. To date, research has demonstrated that mutations of two genes, *MLC1* and *HepaCAM* (also known as *GlialCAM*), are associated with the development of several forms of MLC: the MLC1 type (MIM #604004), the recessive MLC2A type (MIM #613925), and the remitting and dominant MLC2B type (MIM #613926) [2]. Similar to findings observed in patients with MLC, *Mlc1*- and *GlialCAM-*null mice exhibit increased wet weight and normal dry weight of the brain, in addition to progressive white matter vacuolation and intramyelinic edema. Astrocytes from such mice exhibit regulatory volume decrease (RVD) and reduced levels of volume-regulated anion channels (VRACs) [4, 5]. Recent studies have identified a functional network among MLC1, GlialCAM, and ClC-2: GlialCAM targets MLC1 and ClC-2 to specialized glial domains and modifies the biophysical function of ClC-2 in oligodendrocytes. MLC1 is also important for proper localization of GlialCAM and ClC-2, as well as changes in ClC-2 currents [6]. However, the molecular function of MLC1 itself remains to be fully elucidated.

Interestingly, although major causative mutations of MLC are found in the *MLC1* gene, which is expressed specifically in astrocytes, the abnormal phenotypes are observed mainly in oligodendrocytes [4, 6]. Astrocytic dysfunctions have been shown to result in abnormal myelin structure and leukodystrophy in other cases as well. For example, mutations in the glial fibrillary acidic protein (GFAP) gene and the eukaryotic translational initiation factor 2B (EIF-2B) gene lead to Alexander’s disease and vanishing white matter disease, respectively [7, 8]. In addition, astrocytes promote myelin formation by secreting cytokines and growth factors [9] and form heterotypic interactions with OLs via gap junctions [10–12]. These findings suggest that *MLC1* mutation in astrocytes are associated with pathogenic alterations in oligodendrocytes in patients with MLC, which then destabilize interactions between astrocytes and oligodendrocytes and disrupt astrocyte-assisted homeostasis. Indeed, previous studies have demonstrated that stabilization of contact between communicating cells is important for astrocytic regulation of ion and water homeostasis in the brain [12].

During the early stages of cell–cell contact, cell migration should be slowed or paused to ensure appropriate translocation of cell adhesion molecules and the stabilization of cellular interactions. In moving cells, filopodia and lamellipodia formation occur in response to environmental factors, aiding in the regulation of cell migration. Lamellipodia are broad, transient, sheet-like membrane protrusions at the leading edge that play a key role in driving cell migration. In vivo studies have revealed that lamellipodia formation occurs in a variety of cell types during migration [13, 14]. Thus, inhibition of lamellipodia formation may be critical for delaying cell migration and initiating cell–cell contact. In a previous study, the authors used electron microscopy to identify branched actin filament networks in lamellipodia [15]. Moreover, the nucleation of actin branches and elongation of actin filaments are known to generate the major driving force for lamellipodia formation [16–18]. Components of the actin-related protein 2/3 (ARP2/3) complex are essential for the initiation of actin polymerization and the organization of actin networks [19]. The ARP2/3 complex nucleates new (daughter) filaments from the sides of existing (mother) filaments in response to stimulation by nucleation promoting factors (NPFs) such as Wiskott–Aldrich syndrome protein (WASP), suppressor of cyclic AMP repressor (SCAR; also known as WASP-family verprolin-homologous protein (WAVE)), and WASP and SCAR homologue (WASH) complex [19–24]. Additionaly, CORTACTIN is associated with actin dynamics at the leading edge of migrating cells by activating ARP2/3 complex and stabilizing new actin branch points [25, 26]. CORTACTIN-induced actin branching implies that modulation of interaction between ARP2/3 complex and CORTACTIN may be related to regulation of actin dynamics.

In the present study, we demonstrated that MLC1 regulates cellular morphology and motility via remodeling of the actin cytoskeleton: Localization of MLC1 protein at the plasma membrane induced filopodia formation and compensatory decreases in lamellipodia formation, greatly reducing cellular motility and promoting/stabilizing cell–cell interactions. Our findings provide evidence for a functional correlation between surface expression of MLC1 and actin dynamics, suggesting that the pathogenesis of MLC is associated with unstable cell–cell communication and disturbances in ion and water homeostasis.

## Results

### MLC1 regulates morphological changes via remodeling of the actin cytoskeleton

**Fig. 1:**
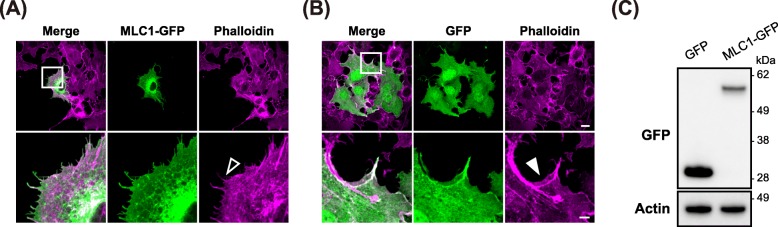
MLC1 induces morphological changes. COS-7 cells were transiently transfected with MLC1-GFP (**a**) or GFP (**b**), followed by immunofluorescence staining with anti-GFP (*green*, transfection marker) antibody and phalloidin (*magenta*, fibrous actin). *Empty* and *filled* arrowheads (magnified image) indicate fibrous actin bundles and branched actin networks, respectively. Scale bar: 20 μm (*upper*) and 5 μm (*bottom*). **c** Expression of MLC1-GFP in COS-7 cells was confirmed via Western blotting with anti-GFP antibody

To investigate the functional role of MLC1, we firstly analyzed the effect of MLC1 over-expression in the heterologous expression system (COS-7 cells). The human MLC1 was fused with GFP to monitor its expression and distribution patterns. Interestingly, the cells expressing MLC1-GFP showed increased spiky membrane protrusions compared to the GFP-expressing cells. Phalloidin staining indicates that the MLC1-GFP expression induces the structural changes of filamentous actin (F-actin): Filopodia formation was increased, but lamellipodia structures were diminished (Fig. 1a, b). The expression of MLC1-GFP proteins in the transfected cells was confirmed by immunoblotting with GFP-specific antibody (Fig. 1c). To eliminate the possibility that the morphological changes were caused by a large GFP-tag itself or perturbation of any cell signaling by the protein tagging at the C-terminal end of MLC1, we examined the effect of MLC1 with a relatively small tag (Myc) on the putative loop between predicted transmembrane domain 3 and 4. The morphological changes were replicated in the cells expressing Myc-tagged MLC1, suggesting that the observed morphological change was not induced by GFP tag, but caused by the expression of MLC1 (Additional file 1: Figure S1).

**Fig. 2:**
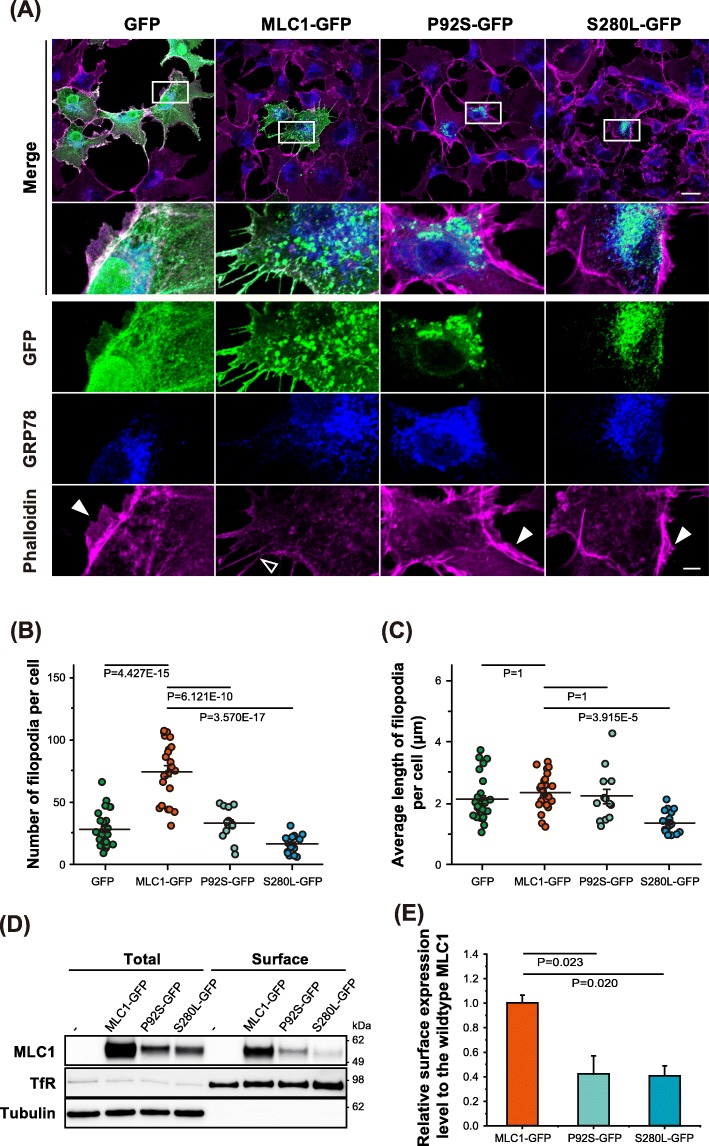
Effect of patient-derived MLC1 mutants on cellular morphology. **a** Heterologously expressed GFP, MLC1-GFP, and patient-derived mutants (P92S- and S280 L-GFP) in COS-7 cells were stained with anti-GFP antibody (*green*, MLC1), GRP78 (*blue*, ER), and phalloidin (*magenta*, fibrous actin). *filled* and *empty* arrowheads indicate branched actin networks and fibrous actin bundles, respectively. Scale bar: 25 μm (*upper*) and 5 μm (*bottom*). The number of filopodia per cell (**b**) and average length of filopodia (**c**) in cells transfected with GFP (*n* = 27 cells and 763 filopodia), MLC1-GFP (*n* = 24 cells and 1788 filopodia), P92S-GFP (*n* = 13 cells and 425 filopodia), and S280 L-GFP (*n* = 17 cells and 281 filopodia) were analyzed by FlioQuant. **d** Surface expression of wildtype and mutant MLC1 in COS-7 cells was analyzed via surface biotinylation (total = 10% of surface). Tubulin was used as a loading control and a cytosol-specific marker. Transferrin receptor (TfR) was used as a surface-specific marker. **e** Densitometric analysis of surface MLC1 expression for wildtype and mutant constructs. Levels of fractional surface expression (surface expression/total protein expression) of tested MLC1 constructs (*n = 3*) were normalized to that of wildtype value (*n* = 3)

Since the previous studies had reported that the MLC1 mutants found in MLC disease patients are mainly localized in subcellular organelles [27–29], we wondered whether patient-derived MLC1 mutants could affect actin remodeling near the plasma membrane. To test the effect of mutant MLC1, we examined morphological changes in COS-7 cells expressing either wildtype or mutant MLC1 proteins (P92S and S280 L) tagged with GFP. As previously reported, two tested mutant MLC1 proteins were mainly trapped in the intracellular organelles, especially colocalized with the ER-specific marker, GRP78 (Fig. 2a) and partially co-localized with the *cis*-Golgi matrix protein, GM130 (Additional file 2: Figure S2A). Interestingly, unlike to the wildtype MLC1, the cells expressing patient-derived mutants failed to exhibit morphological changes in the plasma membrane: Lamellipodia remained intact, and a dense actin network was observed (Fig. 2a). To quantify the morphological changes of the cells expressing either wildtype or mutant MLC1, the total number of filopodia per cell was counted. The number of filopodia was three times higher in wildtype MLC1-expressing cells than in GFP-expressing control cells; however, the filopodia number of the P92S and S280 L mutant MLC1 was decreased to 43.9 and 22.2% of the wildtype, respectively (Fig. 2b). In contrast, filopodia length was not altered by the expression of either wildtype or mutant MLC1, indicating that MLC1 may not be involved in the F-actin elongation process, but in the branching of actin filaments (Fig. 2c).

Since P92S and S280 L were mainly localized at the perinuclear membrane with only a small fraction reaching the plasma membrane (Fig. 2a), we further examined the ability of wildtype and mutant MLC1 proteins for targeting to the plasma membrane. The endogenous transferrin receptor (TfR) was also examined as a plasma membrane marker. Similar to the previous studies, the total expression level of P92S and S280 L mutant was decreased to 50.1 and 42.5% of the wildtype, respectively (Additional file 2: Figure S2B) [27, 28]. In addition, surface biotinylation assay showed that the fractions of MLC1 mutant proteins reaching to the plasma membrane relative to their total protein expressions were significantly decreased compared to the wildtype MLC1 (Fig. 2d, e; the relative surface expression level of P92S and S280 L mutant was decreased to 42.1 and 40.6% of the wildtype, respectively). Interestingly, the severity of deceasing filopodia numbers is somewhat correlated with the degree of decreasing surface expression levels of MLC1 proteins (Fig. 2b, e). These results suggest that the localization of MLC1 at the plasma membrane may be linked to the filopodia formation via actin remodeling.

### MLC1 regulates cellular motility

**Fig. 3:**
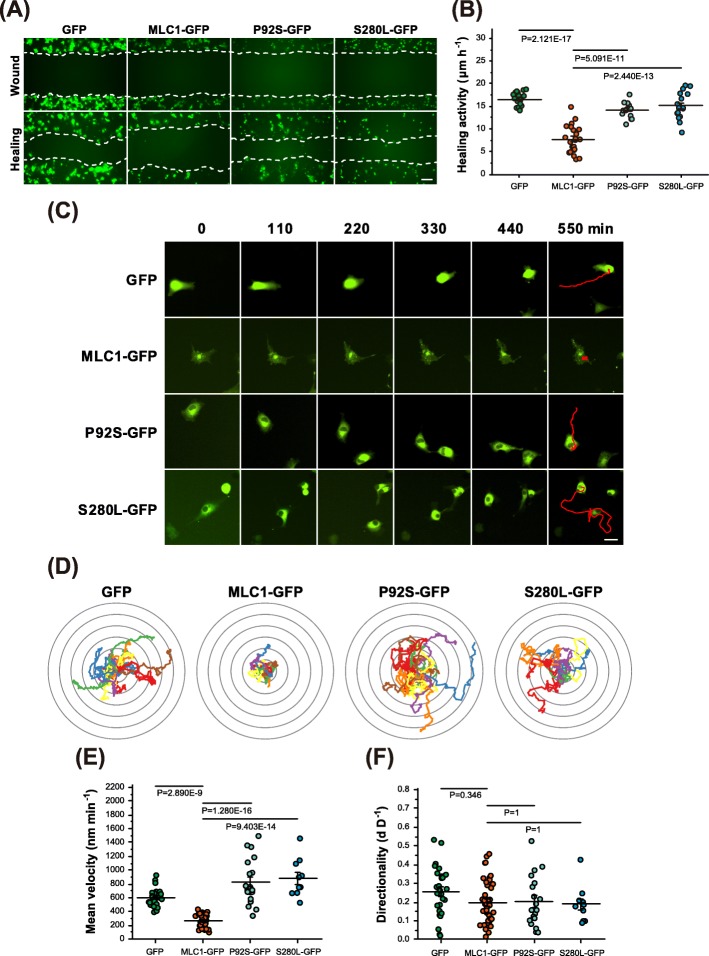
Reduced single-cell motility in an MLC1-expressing cell. **a** Representative images from a wound healing assay of COS-7 cells expressing wildtype MLC1 and patient-derived mutants (P92S- and S280 L-GFP). COS-7 cells expressing GFP-tagged proteins were visualized by 488 nm filter set. Scale bar: 200 μm. **b** Quantification of wound healing activity ((width of initial wound–width of final wound)/24 h) of cells expressing GFP (*n* = 19), MLC1-GFP (*n* = 22), and patient-derived mutants (P92S-GFP (*n* = 16) and S280 L-GFP (*n* = 17)). **c** Representative snapshots and trajectories of single-cell level live cell imaging. Scale bar: 50 μm. Live cell imaging was used to analyze single-cell motility in COS-7 cells expressing GFP (*n* = 27), MLC1-GFP (*n* = 40), and patient-derived mutants (P92S-GFP, *n* = 19 and S280 L-GFP, *n* = 10). Trajectory (**d**), mean velocity (**e**), and directionality (**f**) were analyzed as described in the Methods section. Lower directionality scores are indicative of more random movement

Since actin remodeling, especially through the inhibition of actin branching, is well known to be associated with both cellular morphology and motility [16, 30, 31], we investigated the effect of MLC1 on collective cellular motility by carrying out a wound healing assay. Interestingly, the wildtype MLC1-GFP expressing cells exhibited significant decreases in wound healing activity, however, the mutant (P92S and S280 L) MLC1-expressing cells exhibited normal movement compared to the cells expressing GFP alone (Fig. 3a, b). Since the wound healing assay was performed with transiently transfected cells, the untransfected cells may interfere with the movements of transfected cells with wildtype or mutant MLC1. Also, we cannot exclude the the negative effect of MLC1 on cell proliferation [32], which might affect cell-to-cell interaction and group migration. Thus, we examined the MLC1-dependent cell motility at the single-cell level by taking time-lapse images of live cells over 550 min. As observed in the wound healing assay, only the cell expressing wildtype MLC1 showed significantly decreased cellular motility. The trajectory of wildtype MLC1-expressing cells exhibited restricted boundaries, while those with MLC1 mutants and GFP-expressing cells were instead extended (Fig. 3c, d; Additional file 5: Video S1, Additional file 6: Video S2, Additional file 7: Video S3, Additional file 8: Video S4). The mean velocity of wildtype MLC1-expressing cells was significantly reduced by more than 2-fold (44.2% of the GFP control), but it was not significantly affected by the expression of either P92S or S290 L mutant (Fig. 3e). However, the directionality of movement appeared to be random in all cases (Fig. 3f). Thus, both the collective cell migration and single-cell motility results indicate that subcellular localization of MLC1 can significantly affect cell motility as well as morphological alterations.

### Subcellular localization of MLC1 can alter cellular morphology and motility

**Fig. 4:**
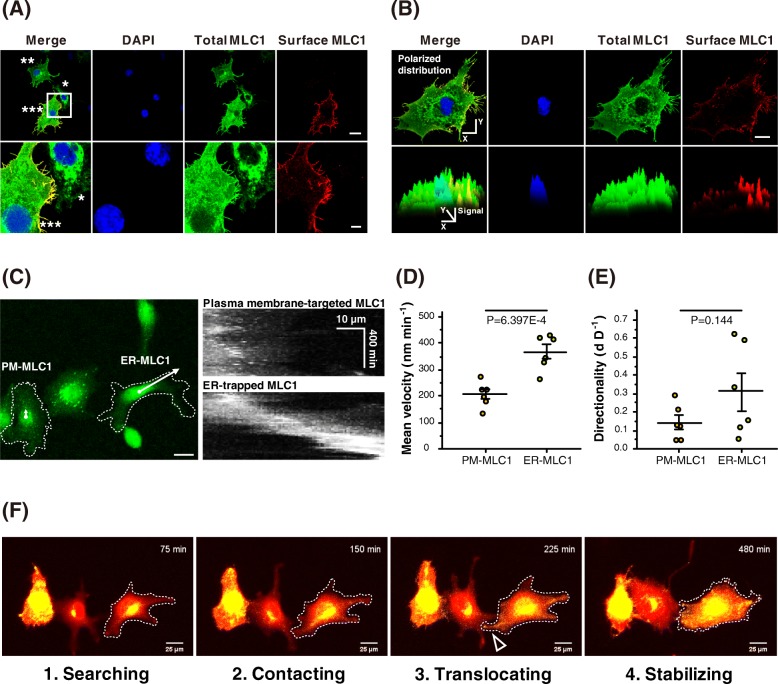
Cellular morphology and motility are regulated by the subcellular localization of wildtype MLC1. **a** Representative image of COS-7 cells expressing MLC1-GFP either in the intracellular organelles (*) or on the plasma membrane (***). COS-7 cell showing intermediate distribution pattern of MLC1 is located on the top of the image (**). Plasma membrane-targeted MLC1 was stained using anti-Myc antibody (*red*, surface MLC1, before permeabilization). Total MLC1 expression was visualized using GFP (*green*, total MLC1, after permeabilization), while nuclear expression was examined using DAPI (*blue*). Scale bar: 20 μm (*upper*), 5 μm (*bottom*). **b** The polarized distribution of surface MLC1-GFP and filopodia. The histogram for each signal was analyzed using ImageJ. Scale bar: 20 μm. **c** Kymographs of COS-7 cells expressing PM-MLC1 and ER-MLC1. Scale bar: 20 μm. Motility of cells expressing PM-MLC1 (*n* = 6) or ER-MLC1 (*n* = 6), as determined via live cell imaging. Mean velocity (**d**) and directionality (**e**) were analyzed. **f** Time-lapse imaging of the subcellular localization of MLC1-GFP in moving COS-7 cells (*yellow*, merged with mCherry). mCherry (*red*) was used as a marker of transfection, allowing us to determine cellular morphology using a fluorescence microscope. Scale bar: 25 μm

To ensure proper targeting of wildtype MLC1 at the plasma membrane, overexpressed MLC1 should escape from the ER and Golgi body over the time course. Thus, we examined whether the subcellular localization of wildtype MLC1 can affect alterations in both morphology and motility during the surface-targeting process. Indeed, some cells transfected with wildtype MLC1 also exhibited mutant-like intracellular localization of MLC1 proteins. From the randomly selected regions of interest (ROIs), 17.5% (*n* = 30) of cells showed that the wildtype MLC1 proteins was trapped intracellularly with a small fraction of surface expression at 24 h after transfection. We classified MLC-expressing cells based on the subcellular distribution of MLC1 proteins: PM-MLC1 cells, in which MLC1 is found at the plasma membrane, and ER-MLC1 cells, in which the significant fraction of MLC1 proteins are trapped in the ER. To detect surface-expressed MLC1 accurately, we placed the Myc-tag in the putative extracellular loop between the third and the fourth transmembrane domains. Immunofluorescence staining confirmed the exclusive accessibility of the Myc-tag from the extracellular side (Additional file 3: Figure S3). Interestingly, the PM-MLC1 cells exhibited abnormal spiky membrane protrusions with the significant surface expression of MLC1 (Fig. 4a, marked with triple asterisks), while the ER-MLC1 cells exhibited normal morphology with feeble MLC1 signals at the cell surface (Fig. 4a, an asterisk), which is somewhat similar to cells expressing patient-derived mutants (Fig. 2a). An intermediate distribution of MLC1 with weak surface MLC1 signals and relatively few filopodia was also observed (Fig. 4a, double asterisks). Moreover, the polarized distribution of surface MLC1 resulted in asymmetric filopodia formation: The region with higher surface expression of MLC1 induced well-developed filopodia formation while reduced surface expression could not. These asymmetric filopodia formation indicates that localization of MLC1 at the plasma membrane is critical to the morphological changes of the cell membrane (Fig. 4b). We also compared cellular motility between PM- and ER-MLC1 cells via live-cell imaging over 500 min. Kymographs of the PM- and ER-MLC1 cells indicated that cellular motility is regulated by the subcellular localization of MLC1 (Fig. 4c). The mean velocity of ER-MLC1 cells was significantly higher than that of PM-MLC1 cells (Fig. 4d), although no changes in directionality were observed as in the cases of patient-derived MLC1 mutants (Fig. 4e). The live-cell imaging allowed us to gain an insight to the functional correlation between subcellular localization of MLC1 and cellular motility. Freely moving cells began to express MLC1 at the initial stage (at 75 min, searching stage), migrated to the target cell, and formed cell-cell contact (at 150 min, contacting stage). After contact, MLC1 translocated from intracellular compartments to the contact point on the plasma membrane (*empty* arrowhead at 225 min, translocating stage). Finally, filopodia formation was increased at the cell-cell contact point, and interactions remained stable for the remainder of the imaging period (at 480 min, stabilizing stage) (Fig. 4f and Additional file 9: Video S1). These results indicate that the translocation of MLC1 stabilizes cell-cell contact by decreasing in cell motility.

### MLC1 acts as a negative regulator of actin branching

**Fig. 5:**
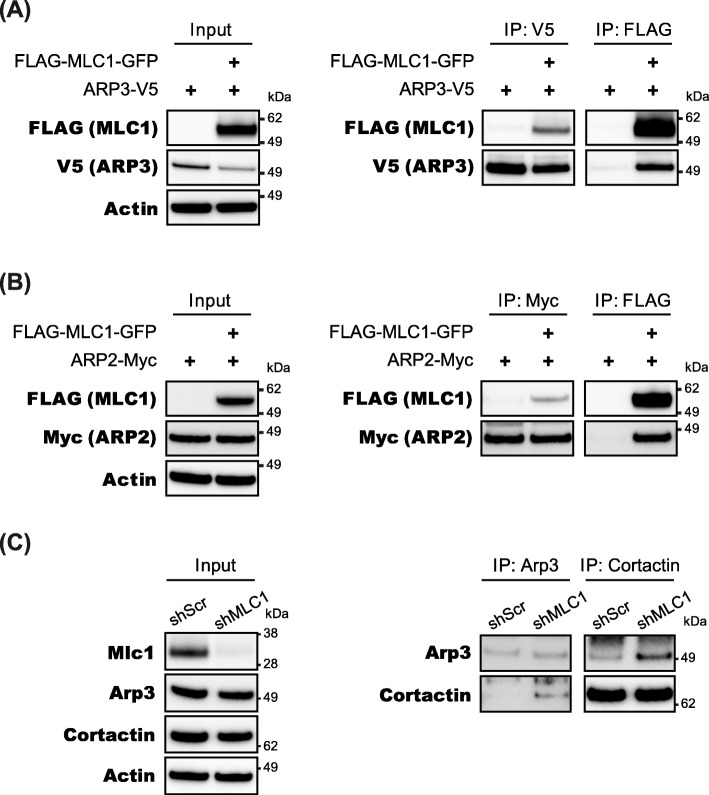
Interaction between MLC1 and ARP2/3 complex. **a** and **b** FLAG-MLC1-GFP, ARP3-V5, and ARP3-Myc were co-expressed in COS-7 cells and used for co-immunoprecipitation. MLC1 and ARP2 or 3 were immunoprecipitated with anti-FLAG, V5, Myc antibodies, respectively. MLC1 was detected in V5- and Myc-precipitants, additionally, ARP3 and ARP2 were detected in FLAG-precipitant. Actin was used as loading control. **c** Primary astrocytes were infected with rAAV-GFP-shScr or -shMlc1. Endogenous Arp3 and Cortactin were immunoprecipitated with specific antibodies (Input = 10% of IP)

**Fig. 6:**
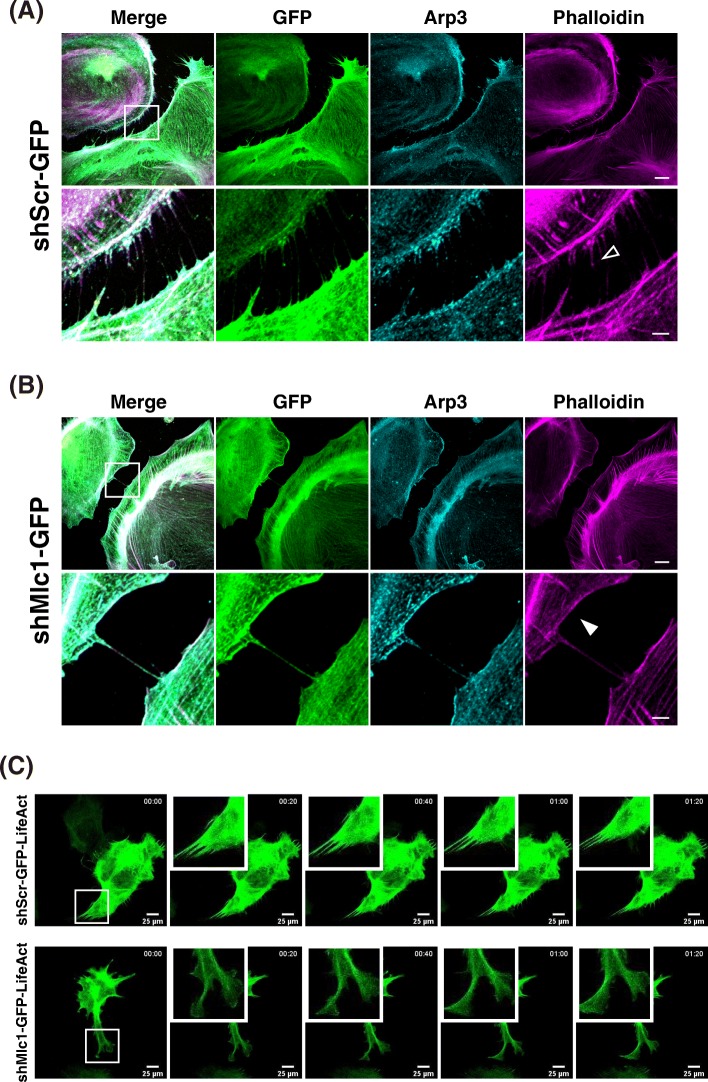
Downregulation of Mlc1 induced lamellipodia formation and dynamic membrane fluctuation in primary astrocytes. Primary astrocytes infected with rAAV encoding GFP and shScrambled Mlc1 (shScr, **a**) or shMlc1 (**b**) were stained with anti-GFP (*green*, shRNA-transfected), Arp3 (*cyan*), and phalloidin (*magenta*) antibodies. In the magnified cell-cell contact points (*bottom*), fibrous actin bundles and branched actin networks are indicated by *empty* and *filled* arrowheads, respectively. Scale bar: 20 μm (*upper*) and 5 μm (*bottom*). **c** Time-lapse imaging of primary astrocytes expressing GFP-LifeAct and shScr or shMlc1. Scale bar: 25 μm

How does the surface expression of MLC1 induce the morphological changes of the cell via actin cytoskeletal remodeling? Previous studies reported that inhibition of the actin-related protein, ARP can induce filopodia formation and reduce lamellipodia formation by inhibiting the branching of actin filaments, thereby decreasing cellular motility [30]. Since the ARP2/3 complex is a known indicator of actin branching points [18, 24] and CORTACTIN is known as stabilizer of actin branch [25, 26], we wondered whether the MLC1 at the plasma membrane could perturb the branching of actin filament and inhibit lamellipodia formation. Thus, we tested whether MLC1 proteins could interact with ARP2/3 complexes in the COS-7 cells. Indeed, MLC1 can interact with both ARP2 and ARP3 (Fig. 5a, b). We had tried to replicate MLC1-ARP2/3 complex interaction in the primary astrocytes, but commercial antibody against MLC1 failed to immunoprecipitate MLC1 protein in primary astrocyte lysate. Then, we tested whether MLC1 proteins could affect the interaction between ARP3 and Cortactin in the primary astrocytes. Immunoprecipitation assay with anti-ARP3 or Cortactin antibody revealed that binding of ARP3 to Cortactin was enhanced by downregulation of Mlc1(Fig. 5c). These results indicate that Mlc1 may act as a negative regulator of actin branching and lamellipodia formation. These results inspired us to test the effect of Mlc1 knockdown on the actin remodeling and subcellular localization of Arp3 in the S100β-positive matured primary astrocytes. Before examining the effect of Mlc1 knockdown, the efficiency of Mlc1-specific shRNA (shMlc1) was evaluated in the mouse primary astrocytes with recombinant AAV (rAAV) encoding both shMlc1 and GFP. Infection with shMlc1 virus significantly reduced the endogenous Mlc1 expression level in the primary astrocyte culture, which indicated that shMlc1 is useful to silence Mlc1 (Additional file 4: Figure S4A). Interestingly, Arp3 was co-localized with fibrous actin bundle (Fig. 6a) and dense actin network (Fig. 6b) in shScr- and shMlc1-infected primary astrocytes, respectively. Immunofluorescence staining showed that knockdown of Mlc1 repressed filopodia formation but induced lamellipodia formation as indicated by Phalloidin staining pattern (in both of AraC-treated astrocyte culture (Fig. 6b) and canonical astrocyte culture (Additional file 4: Figure S4C)). Taken together, these results indicate that Mlc1 does not affects subcellular localization of Arp3 but may competes with Cortactin for interaction with Arp3 resulting in reduced stability of actin branch and morphological change.

Additionally, we found that MLC1 stabilizes membrane structure of primary astrocytes. The real-time image of shMlc1-transfected primary astrocytes showed dynamic membrane ruffling, which indicated that actin filament reorganization has actively occurred. Instead, the membrane structures are somewhat static in the primary astrocytes transfected with shScr (Fig. 6c; Additional file 10: Video S1 and Additional file 11: Video S2). Collectively, these results indicate that the expression of Mlc1 may interferes branching of actin filament and stabilization of membrane fluctuation.

## Discussions

Actin filaments provide cells with mechanical support, track intracellular trafficking, and provide the driving force for cell movement, all of which are critical to cellular physiology [31]. Various proteins are involved in the regulation of actin dynamics, playing key roles in the maintenance of monomeric actin molecules, the nucleation and elongation of actin filaments, branch formation, filament–filament interactions, and the delivery of cargo. Among these proteins, the ARP2/3 complex is well known to aid in the formation of branched actin filaments [19]. In the present study, we investigated the molecular function of MLC1 and its effects on cell–cell interactions. We observed that MLC1 proteins compete with Cortactin for interaction with the ARP2/3 complex, thereby decreasing the formation of branched actin filaments. In addition, mutant MLC1 proteins (P92S and S280 L) were associated with reduced levels of expression and plasma membrane targeting (Fig. 2d, e). Such decreases in the expression of mutant MLC1 may be related to altered protein stability, as suggested in previous studies [27, 28]. These results suggest that MLC1 may inhibit actin branching by disturbing interactions between the ARP complex and actin nucleation factors near the plasma membrane. This hypothesis is supported by the strong correlation between plasma membrane targeting of wildtype MLC1 over the course of subcellular localization and its effects on cellular morphology and motility (Fig. 4).

MLC1 protein is known to be highly expressed in primary astrocytes, as well as in lymphocytes and monocytes [33, 34]. Petrini et al. demonstrated that MLC1 in monocytes was distributed from the perinuclear area to the filopodia or podosomes. MLC1 expression was found to be decreased in macrophages isolated from patients with MLC and this was because MLC1 proteins were trapped in the ER. Patient-derived cells also exhibited a reduced ability to adhere to plastic supports and an altered cytoskeletal structure in both actin and tubulin filaments [34]. In this study we replicated the observations, suggesting that MLC1-dependent changes in cellular morphology and motility represent general effects that can be observed regardless of their cellular origins.

In cultured primary astrocytes, the trafficking and membrane expression of MLC1 is known to be regulated by caveolin-mediated trafficking and phosphorylation by protein kinase A (PKA) and protein kinase C (PKC) [35]. Previous studies have attempted to restore expression of MLC1 mutants by facilitating protein folding (e.g., lowering the temperature to 33 °C or treating the cells with glycerol as a chemical chaperone) or decreasing protein degradation using a FDA-approved proteasomal inhibitor (Velcade®) [27]. It was reported that a mutation in *GlialCAM* affects the trafficking of MLC1 [36]. In the heterologous expression system, pathogenic mutant GlialCAM could not accumulate at the cell–cell junction, and MLC1 exhibited a dispersed pattern of expression. Thus, the authors speculated that GlialCAM may act as a carrier protein during membrane trafficking and targeting of MLC1. Interestingly, MLC2B-type MLC is caused by a point mutation of GlialCAM. In patients with this form of the disease, typical clinical symptoms of MLC can be observed in the early stages, although they may improve thereafter [37]. Such findings suggest that other mediators of MLC1 trafficking can compensate for the actions of GlialCAM. Since our data provide evidence for the role of MLC1 in actin dynamics, regulating actin filaments and modulating protein stability/surface trafficking of MLC1 may aid in the treatment of MLC.

The *MLC1* gene was initially identified as a putative cation channel purely based on amino acid sequence alignment [38]. However, electrophysiological analyses revealed that MLC1 did not directly mediate any ionic currents [39]. Previous studies have reported that MLC1 can form a multi-protein complex with various membrane transport proteins including Kir4.1, AQP4, TRPV4, and the β-subunit of Na^+^/K^+^-ATPase in cultured primary astrocytes [29, 40]. Functional studies have indicated that mutations of MLC1 attenuate TRPV4-mediated Ca^2+^ influx and RVD-induced Cl^−^ currents upon hypoosmotic stimulation [29, 41]. A recent study reported that reduced volume-regulated anion current and impaired RVD are related to MLC1 and LRRC8A, the main subunit of VRACs. Downregulation of either MLC1 or LRRC8A decreases the VRAC current [42]. In addition, research has demonstrated that developmental myelination and myelin maintenance are normal in *Mlc1*-null and *GlialCAM*-null mice [4]. In this report, intrinsic neuronal excitability remained unaltered in both MLC mouse models. However, the extracellular K^+^ concentration increased upon network activation due to the dysfunction of astrocytic MLC1, thereby disturbing [K^+^]_o_ dynamics and leading to network hyperexcitability. These data indicate that MLC1 may regulate other ion channels instead of forming an ion channel itself. However, we cannot exclude the possibility that MLC1 directly or indirectly affect a membrane transport function that permeates a solute other than Ca^2+^ or Cl^−^. The previous study did not suggest any relationship between TRPV4-mediated Ca^2+^-influx and actin remodeling [29]; however, it is worth noting that increased intracellular calcium concentration can stabilize postsynaptic F-actin via calcium sensor caldendrin and lead to transient and global reorganization of actin filament [43, 44].

Because our results indicate that MLC1 is important for the regulation of cellular motility as well as stable cellular communication, the impairments in oligodendrocyte volume regulation observed in patients with MLC and mouse models may be related to unstable communication between oligodendrocytes and astrocytes caused by MLC1 dysfunction. Since the surface area of astrocyte should respond to the hypotonic challenges, increased filopodia formation induced by surface expression of MLC1 may serve as a membrane reservoir in astrocytes that can readily respond to the astrocytic volume increase. However, the detailed molecular mechanisms underlying MLC1-induced alterations in morphology and motility remain to be elucidated. The proteomic analysis for identifying proteins differently interacting with wildtype or mutant MLC1 would deepen our understanding how the actin-cytoskeltal remodeling can be induced by the expression of a multi-passing membrane protein, MLC1 in the plasma membrane. It will be interesting to see whether wildtype and pathogenic mutants take a distinct phosphorylation status and their effects on the morphological and motility changes of astrocyte. Also, how the astrocytic morphology and motility changes induced by the *MLC1*-mutations are linked to the pathological outcomes of MLC disease warrants further investigation in vivo.

In summary, the results of the present study demonstrate that the membrane protein MLC1 modulates cellular morphology and motility in a subcellular targeting-dependent manner: Expression of MLC1 protein at the plasma membrane promotes filipodia formation and decreases cellular motility, neither of which occurs when it is expressed in intracellular organelles. Our findings provide new insight into the pathogenesis of astrocyte dysfunction-induced forms of leukodystrophy, especially MLC disease.

## Methods

### Cell cultures

The COS-7 cell line was obtained from ATCC (CRL-1651™) and maintained in Dulbecco’s modified Eagle’s medium (DMEM) supplemented with 10% fetal bovine serum (Thermo) and 100 U/mL penicillin–streptomycin (Thermo) at 37 °C in a 5% CO_2_ incubator. Primary astrocytes were isolated from 0~2-day-old newborn C57BL/6 mouse (KOATECH). Isolation of pure astrocytes was performed as previously described with slight modifications [45, 46]. Briefly, the cerebral cortices were dissected, and the meninges were carefully removed. Tissue samples were cut into small pieces using sharp blades and transferred to Hank’s balanced salt solution (HBSS) supplemented with 20 mM HEPES (pH 7.3) (Thermo), 1 mM sodium pyruvate (Thermo), 1X antibiotic–antimycotic solution (Thermo), 0.03% Trypsin/EDTA (Thermo), and 50 U/mL DNase I (Roche). After incubation at 37 °C for 15 min, tissues were triturated using a fire-polished Pasteur pipette. Dissociated cells were passed through a cell strainer (70 μm) and plated on a T75 flask per 3 pups. Astrocytes were further maintained in DMEM supplemented with 1X GlutaMAX (Thermo), 1 mM sodium pyruvate, 10% fetal bovine serum (Thermo), and 100 U/mL penicillin–streptomycin (Thermo) at 37 °C in a 5% CO_2_ incubator. To prevent oligodendrocyte precursor cell contamination, the flask was vigorously shaken by hand for 1 min when cells were confluent. To test diverse culture methods, parallel culture was supplemented with 2 μM Cytosine β-D-arabinofuranoside (AraC, Sigma) as previously recommended [47]. Cells were seeded on poly-L-lysine-coated coverslips or confocal dishes for immunofluorescence staining or live cell imaging, respectively. At DIV 21–25, maturation of astrocytes was examined by S100β staining (Additional file 4: Figure S4) and used for assays. The experimental protocol was approved by the Animal Care and Use Committee of Korea Brain Research Institute (IACUC-2018-0013).

### Transfection and transduction

Cells were plated 1 day prior to transfection at 50–60% of confluence and transfected using a calcium phosphate transfection kit (Thermo) according to the manufacturer’s instructions. A total of 1.5 μg of DNA was added to the 35-mm culture dish for each reaction. To transfect, confluent primary astrocytes were seeded in culture vessels at DIV 14 and transfected as described above. Primary astrocytes were transduced with rAAV at a multiplicity of infection (MOI) of 10 to reduce levels of Mlc1 expression. Transduction efficiency was estimated by analyzing level of GFP expression. Plasmid and rAAV vector containing both the green fluorescent protein (GFP) gene and Mlc1-specific shRNA (shMlc1, 5`-GGAGAAATGTCAGTGCGATTC-3`) were obtained from VectorBuilder (www.vectorbuilder.com).

### Plasmid construction

Total RNA from primary astrocytes was purified using a RNeasy Mini kit (Qiagen), while single-strand cDNA was synthesized using a QuantiTect Reverse Transcription Kit (QIAGEN), in accordance with manufacturer instructions. Mouse (NM_133241.2) and human (NM_015166.3) MLC1 were obtained from GenScript. Complementary DNA of other constructs was amplified using Q5 polymerase (NEB) with primers flanking the restriction enzyme sites: ARP3 (NM_NM_005721.4); 5`-ACGGTACCGCCACCATGGCGGGACGGCT-3` and 5`-GCCGCTAGCCGACATGACTCCAAACACTGGATTGT-3`, ARP2 (NM_005722.3); 5`-GACGGTACCGCCACCATGGACAGCCAGGGCAG-3` and 5`-TCACCGGTTCGAACAGTCACACCAAGTTTCTC-3`. Patient-derived mutants were generated via site-directed mutagenesis (Agilent). Purified polymerase chain reaction (PCR) products were inserted into a mammalian expression vector under the control of the CAG promoter.

### Antibodies and fluorescence-labeled probes

Antibodies against MLC1 and ARP3 were obtained from Abcam (catalog number: ab130770 and ab49671, respectively). Antibodies against Myc and ARP2 were obtained from Cell Signaling (catalog number: 2278S and 3128S, respectively), and those against GFP and V5 were obtained from Thermo (catalog number: A10262 and R960–25, respectively). Anti-mouse and anti-rabbit horseradish peroxidase (HRP)-conjugated secondary antibodies were obtained from GenDEPOT. Fluorescence-conjugated secondary antibodies for anti-mouse, anti-rabbit, and anti-chicken immunoglobulin G were obtained from Thermo. Fluorescence-conjugated phalloidin was obtained from Thermo.

### Western blotting

Cells were lysed in lysis buffer (1X PBS, 1% (v/v) Triton-100, and 1X protease inhibitor cocktail, Roche) and gently inverted for 1 h at 4 °C. Cell debris were removed via centrifugation and the cleared lysate was mixed with final 1X LDS sample buffer (Thermo). Samples were separated in 4–12% Bis-Tris Plus gel (Bolt, Thermo) and the separated proteins were transferred to polyvinylidene fluoride (PVDF) membranes using an iBlot2 Gel Transfer Device (Thermo). To suppress the non-specific binding of antibodies, the membranes were incubated with the blocking solution (1X PBS, 0.1% Tween-20, and 1% (w/v) bovine serum albumin) for 1 h at room temperature. Proteins were detected by incubating membrane with the primary antibodies (0.25–1 μg/mL in blocking solution) for 1~2 h and followed HRP-conjugated secondary antibodies (0.1–0.5 μg/mL in blocking solution) for 1 h at room temperature. The protein bands were visualized by the electrochemiluminescence (ECL) reactions and the images were taken by using ChemiDoc MP (BIO-RAD).

### Wound healing assay

COS-7 cells transiently transfected with GFP, MLC1-GFP, and patent-derived mutants (P92S-GFP and S280 L-GFP) were maintained until confluent. Wounds were made by scratching the cell mono-layer using 1 mL pipette tip and suspended cell were removed by intensive wash with complete media. After 24 h, healing activity was determined by dividing the subtracted distance between leading edge of wound (width of initial wound-width of final wound) with total healing time (24 h).

### Immunoprecipitation assay

Transfected cells were harvested and lysed in lysis buffer (1% (v/v) Triton X-100, 1X PBS, 1X protease inhibitor cocktail, Roche) for 1 h at 4 °C and centrifuged to remove cell debris. Cell lysates were pre-cleared by incubating with an equal volume of PBS containing Protein G beads (10 μL of 50% slurry per 200 μL lysis buffer, GenScript). Pre-cleared cell lysates were transferred to the antibody–bead mixture (0.5 μg per 10 μL beads) and incubated overnight at 4 °C with gentle inverting. Protein of interest (POI)–antibody–bead complexes were washed three times with lysis buffer. Immunoprecipitants were eluted with 2X LDS sample buffer (Thermo) and subjected to Western blotting.

### Immunofluorescence staining and imaging

Cells were seeded on poly-L-lysine (PLL)-coated coverslips and transfected with appropriate constructs. Cells were fixed in a solution containing 4% paraformaldehyde (Electron Microscopy Sciences), 1X PBS, and 0.04 g/mL sucrose for 10 min, following which they were permeabilized with 0.2% (v/v) Triton X-100 in PBS for 10 min. For non-permeabilized condition, primary antibodies (2–10 ng/μL) prepared in detergent-free blocking solution (3% (w/v) bovine serum albumin, 1X PBS) were used before the permeabilizing step. Other primary antibodies were prepared in blocking solution (3% (w/v) bovine serum albumin, 1X PBS, 0.1% Tween-20) and used after permeabilization. After blocking for 1 h, primary antibodies were incubated for 1 h and washed out with washing solution (1X PBS with or without 0.1% (v/v) Tween-20 for permeabilized or non-permeabilized condition). Secondary antibodies were incubated for 45 min and washed out with washing solution. Coverslips were mounted on glass slides using ProLong Gold antifade solution (Thermo) and cured for overnight. Confocal images were obtained using an inverted Nikon ECLIPSE Ti-E confocal microscope equipped with an oil-immersion objective lens (Nikon plan Apochromat 60 X / NA 1.40). Antibody reactions and microscopic imaging were performed at 23 °C. For live cell imaging, cells were seeded on PLL-coated glass-bottom dishes and transfected with appropriate constructs. Live cell images were obtained using a Nikon ECLIPSE Ti-E confocal microscope and Nikon Ti-E live cell microscope equipped with a TI-S-ER motorized stage using a Nikon plan Apo 20X/NA 0.75 objective lens and Nikon Plan Fluor 10X/NA 0.3 objective lens, respectively. Image analysis (quantification of filopodia number / length, determination of velocity / directionality / trajectory, and kymography) was performed using the plugins of ImageJ (FlioQuant, Manual Tracking, and Kymograph, respectively (National Institutes of Health)).

Directionality was calculated as follows:

$$ \mathrm{Directionality}=\frac{\mathrm{d}}{\mathrm{D}} $$

where *d* represents the distance between the starting and ending points of the target cell and *D* represents the total length of the trajectory of the target cell. Confocal and live cell imaging data were acquired at the Brain Research Core Facilities in KBRI (Korea).

### Statistical analysis

Data are presented as the mean ± the standard error of the mean (s.e.m.). No statistical method was used to predetermine the sample size, and no adjustments were made for multiple comparisons. The statistical analyses were performed using Origin 10.6 (OriginLab) and SigmaPlot 13.0 (Systat Software) software packages. One-Way ANOVA test was used for comparisons, and the appropriate *P* values are indicated in each graph.

## Supplementary information

**Additional file 1: Figure S1**. Myc-tagged MLC1 induces morphological changes. (A) Schematic drawings of a putative transmembrane topology of hMLC1 used in this study. For the hMLC1-GFP construct, *GFP* was in-framed fused to the 3′-end of *hMLC1* (*green*). (B) For generating Myc-tagged hMLC1, the Myc-tag was placed in the putative extracellular loop of hMLC1 (*blue flag*). (C) Myc-tagged MLC1 (*cyan*) also induced the filopodia formation (*dotted* box) as MLC1-GFP in transfected cell, however, untransfected cell showed lamellipodia (*solid* box). Fibrous actin was stained with Phalloidin (*magenta*). Scale bar: 25 μm and 5 μm (magnified image).

**Additional file 2: Figure S2**. Partial co-localization of patient-derived mutants and *cis*-Golgi matrix protein. (A) GFP, MLC1 and patient-derived mutants (P92S- and S280 L-GFP) were expressed in COS-7 cells. Wildtype and mutant MLC1 (*green*, GFP) were partially co-localized with GM130 (*yellow*, *cis*-Golgi matrix protein)*.* Fibrous actin was stained with Phalloidin (*magenta*)*.* Scale bar: 20 μm. (B) The total expression levels of MLC1, P92S, and S280 L were normalized to the expression level of Tubulin and quantified.

**Additional file 3: Figure S3**. The antibody accessibility to the Myc-epitope placed in the putative extracellular loop of MLC1. (A) Schematic drawings of a putative transmembrane topology of hMLC1-Myc-GFP. The *Myc*-tag was placed in the putative extracellular loop of hMLC1 (*blue flag*, extracellular) and *GFP* was in-framed fused to the 3′-end of *hMLC1* (*green*, intracellular). (B) For the “Non-permeabilized” condition, Myc epitope in the putative extracellular loop was stained with anti-Myc antibody before permeabilization (*yellow*, PM-MLC1). After permeabilized, cells were stained with anti-GFP antibody (*green*, total MLC1), DAPI, and Phalloidin (*magenta*). For the “Permeabilized” condition, all of staining procedures were performed after permeabilization. Anti-Myc antibody was accessible from both extracellular and intracellular sides only after permeabilization. Scale bar: 25 μm.

**Additional file 4: Figure S4**. Examine the infection efficiency and the effect of MLC1 on the matured primary astrocytes. (A) Knockdown of MLC1 in mouse primary astrocytes was performed by viral infection and was confirmed via Western Blotting with anti-MLC1 antibody. GFP expression levels were analyzed to evaluate the efficiency of AAV infection. Similar GFP expression level in the astrocytes treating with either shMlc1 or the scrambled shMlc1 (shScr) virus indicates that the infection efficiency was similar. Actin was used as loading control. (B and C) To verify the maturation of primary astrocytes, cells were stained with anti-S100β antibody (*magenta*). *Empty* and *filled* arrowheads in the magnified image indicate fibrous actin bundles and branched actin networks, respectively.

**Additional file 5: Video S1A.** Time-laps image (4 frames per hour) of COS-7 cells expressing GFP, wildtype and mutant MLC1 fused with GFP; GFP.

**Additional file 6: Video S1B.** Time-laps image (4 frames per hour) of COS-7 cells expressing GFP, wildtype and mutant MLC1 fused with GFP; hMLC1-GFP.

**Additional file 7: Video S1C.** Time-laps image (4 frames per hour) of COS-7 cells expressing GFP, wildtype and mutant MLC1 fused with GFP; P92S-GFP.

**Additional file 8: Video S1D.** Time-laps image (4 frames per hour) of COS-7 cells expressing GFP, wildtype and mutant MLC1 fused with GFP; S280 L-GFP.

**Additional file 9: Video S2.** Time-laps image (4 frames per hour) was taken to analyze change in subcellular distribution of MLC1 in freely moving COS-7 cells. Snap shot images are presented in Fig. 4f.

**Additional file 10: Video S3A.** Time-laps image (12 frames per hour) was taken to analyze morphological change of primary astrocytes transfected with shScr.

**Additional file 11: Video S3B.** Time-laps image (12 frames per hour) was taken to analyze morphological change of primary astrocytes transfected with shMlc1-GFP-LifeAct.

## Data Availability

The materials and datasets used and/or analyzed during the current study are available from the corresponding author on reasonable request.

## Abbreviations

ARP: actin-related protein
DMEM: Dulbecco’s modified Eagle’s medium
ECL: electrochemiluminescence
EIF-2B: eukaryotic translational initiation factor 2B
GFAP: glial fibrillary acidic protein
GFP: green fluorescent protein
HBSS: Hank’s balanced salt solution
HRP: horseradish peroxidase
MLC: megalencephalic leukoencephalopathy with subcortical cysts
MOI: multiplicity of infection
NPF: nucleation promoting factors (NPFs)
PCR: polymerase chain reaction
PKA: protein kinase A
PKC: protein kinase C
PLL: poly-L-lysine
POI: protein of interest
PVDF: polyvinylidene fluoride
rAAV: recombinant AAV
RVD: regulatory volume decrease
SCAR: suppressor of cyclic AMP repressor
VRAC: volume-regulated anion channels
WASH: WASP and SCAR homologue complex
WASP: Wiskott–Aldrich syndrome protein
WAVE: WASP-family verprolin-homologous protein

## References

[1] van der Knaap MS, Valk J, Barth PG, Smit LM, van Engelen BG, Tortori Donati P (1995). Leukoencephalopathy with swelling in children and adolescents: MRI patterns and differential diagnosis. Neuroradiology.

[2] van der Knaap MS, Boor I, Estevez R (2012). Megalencephalic leukoencephalopathy with subcortical cysts: chronic white matter oedema due to a defect in brain ion and water homoeostasis. Lancet Neurol.

[3] van der Knaap MS, Barth PG, Stroink H, van Nieuwenhuizen O, Arts WF, Hoogenraad F, Valk J (1995). Leukoencephalopathy with swelling and a discrepantly mild clinical course in eight children. Ann Neurol.

[4] Dubey M, Bugiani M, Ridder MC, Postma NL, Brouwers E, Polder E, Jacobs JG, Baayen JC, Klooster J, Kamermans M (2015). Mice with megalencephalic leukoencephalopathy with cysts: a developmental angle. Ann Neurol.

[5] Bugiani M, Dubey M, Breur M, Postma NL, Dekker MP, Ter Braak T, Boschert U, Abbink TEM, Mansvelder HD, Min R (2017). Megalencephalic leukoencephalopathy with cysts: the Glialcam-null mouse model. Ann Clin Transl Neurol.

[6] Hoegg-Beiler MB, Sirisi S, Orozco IJ, Ferrer I, Hohensee S, Auberson M, Godde K, Vilches C, de Heredia ML, Nunes V (2014). Disrupting MLC1 and GlialCAM and ClC-2 interactions in leukodystrophy entails glial chloride channel dysfunction. Nat Commun.

[7] van der Knaap MS, Leegwater PA, Konst AA, Visser A, Naidu S, Oudejans CB, Schutgens RB, Pronk JC (2002). Mutations in each of the five subunits of translation initiation factor eIF2B can cause leukoencephalopathy with vanishing white matter. Ann Neurol.

[8] Brenner M, Johnson AB, Boespflug-Tanguy O, Rodriguez D, Goldman JE, Messing A (2001). Mutations in GFAP, encoding glial fibrillary acidic protein, are associated with Alexander disease. Nat Genet.

[9] Barnett SC, Linington C (2013). Myelination: do astrocytes play a role?. Neuroscientist.

[10] Tress O, Maglione M, Zlomuzica A, May D, Dicke N, Degen J, Dere E, Kettenmann H, Hartmann D, Willecke K (2011). Pathologic and phenotypic alterations in a mouse expressing a connexin47 missense mutation that causes Pelizaeus-Merzbacher-like disease in humans. PLoS Genet.

[11] Lutz SE, Zhao Y, Gulinello M, Lee SC, Raine CS, Brosnan CF (2009). Deletion of astrocyte connexins 43 and 30 leads to a dysmyelinating phenotype and hippocampal CA1 vacuolation. J Neurosci.

[12] Lanciotti A, Brignone MS, Bertini E, Petrucci TC, Aloisi F, Ambrosini E. Astrocytes: emerging stars in Leukodystrophy pathogenesis. Transl Neurosci. 2013;4:144-64. 10.2478/s13380-013-0118-1 PMC3856885 24340223

[13] Friedl P, Gilmour D (2009). Collective cell migration in morphogenesis, regeneration and cancer. Nat Rev Mol Cell Biol.

[14] Weijer CJ (2009). Collective cell migration in development. J Cell Sci.

[15] Svitkina TM, Borisy GG (1999). Arp2/3 complex and actin depolymerizing factor/cofilin in dendritic organization and treadmilling of actin filament array in lamellipodia. J Cell Biol.

[16] Krause M, Gautreau A (2014). Steering cell migration: lamellipodium dynamics and the regulation of directional persistence. Nat Rev Mol Cell Biol.

[17] Ridley AJ, Schwartz MA, Burridge K, Firtel RA, Ginsberg MH, Borisy G, Parsons JT, Horwitz AR (2003). Cell migration: integrating signals from front to back. Science.

[18] Ridley AJ (2011). Life at the leading edge. Cell.

[19] Goley ED, Welch MD (2006). The ARP2/3 complex: an actin nucleator comes of age. Nat Rev Mol Cell Biol.

[20] Pollard TD (2007). Regulation of actin filament assembly by Arp2/3 complex and formins. Annu Rev Biophys Biomol Struct.

[21] Padrick SB, Rosen MK (2010). Physical mechanisms of signal integration by WASP family proteins. Annu Rev Biochem.

[22] Campellone KG, Welch MD (2010). A nucleator arms race: cellular control of actin assembly. Nat Rev Mol Cell Biol.

[23] Machesky LM, Insall RH (1998). Scar1 and the related Wiskott-Aldrich syndrome protein, WASP, regulate the actin cytoskeleton through the Arp2/3 complex. Curr Biol.

[24] Rotty JD, Wu C, Bear JE (2013). New insights into the regulation and cellular functions of the ARP2/3 complex. Nat Rev Mol Cell Biol.

[25] Weaver AM, Karginov AV, Kinley AW, Weed SA, Li Y, Parsons JT, Cooper JA (2001). Cortactin promotes and stabilizes Arp2/3-induced actin filament network formation. Curr Biol.

[26] Weaver AM, Heuser JE, Karginov AV, Lee WL, Parsons JT, Cooper JA (2002). Interaction of cortactin and N-WASp with Arp2/3 complex. Curr Biol.

[27] Duarri A, Teijido O, Lopez-Hernandez T, Scheper GC, Barriere H, Boor I, Aguado F, Zorzano A, Palacin M, Martinez A (2008). Molecular pathogenesis of megalencephalic leukoencephalopathy with subcortical cysts: mutations in MLC1 cause folding defects. Hum Mol Genet.

[28] Teijido O, Martinez A, Pusch M, Zorzano A, Soriano E, Del Rio JA, Palacin M, Estevez R (2004). Localization and functional analyses of the MLC1 protein involved in megalencephalic leukoencephalopathy with subcortical cysts. Hum Mol Genet.

[29] Lanciotti A, Brignone MS, Molinari P, Visentin S, De Nuccio C, Macchia G, Aiello C, Bertini E, Aloisi F, Petrucci TC, Ambrosini E (2012). Megalencephalic leukoencephalopathy with subcortical cysts protein 1 functionally cooperates with the TRPV4 cation channel to activate the response of astrocytes to osmotic stress: dysregulation by pathological mutations. Hum Mol Genet.

[30] Wu C, Asokan SB, Berginski ME, Haynes EM, Sharpless NE, Griffith JD, Gomez SM, Bear JE (2012). Arp2/3 is critical for lamellipodia and response to extracellular matrix cues but is dispensable for chemotaxis. Cell.

[31] Pollard TD, Cooper JA (2009). Actin, a central player in cell shape and movement. Science.

[32] Lanciotti A, Brignone MS, Visentin S, De Nuccio C, Catacuzzeno L, Mallozzi C, Petrini S, Caramia M, Veroni C, Minnone G (2016). Megalencephalic leukoencephalopathy with subcortical cysts protein-1 regulates epidermal growth factor receptor signaling in astrocytes. Hum Mol Genet.

[33] Boor PK, de Groot K, Waisfisz Q, Kamphorst W, Oudejans CB, Powers JM, Pronk JC, Scheper GC, van der Knaap MS (2005). MLC1: a novel protein in distal astroglial processes. J Neuropathol Exp Neurol.

[34] Petrini S, Minnone G, Coccetti M, Frank C, Aiello C, Cutarelli A, Ambrosini E, Lanciotti A, Brignone MS, D'Oria V (2013). Monocytes and macrophages as biomarkers for the diagnosis of megalencephalic leukoencephalopathy with subcortical cysts. Mol Cell Neurosci.

[35] Lanciotti A, Brignone MS, Camerini S, Serafini B, Macchia G, Raggi C, Molinari P, Crescenzi M, Musumeci M, Sargiacomo M (2010). MLC1 trafficking and membrane expression in astrocytes: role of caveolin-1 and phosphorylation. Neurobiol Dis.

[36] Lopez-Hernandez T, Sirisi S, Capdevila-Nortes X, Montolio M, Fernandez-Duenas V, Scheper GC, van der Knaap MS, Casquero P, Ciruela F, Ferrer I (2011). Molecular mechanisms of MLC1 and GLIALCAM mutations in megalencephalic leukoencephalopathy with subcortical cysts. Hum Mol Genet.

[37] Lopez-Hernandez T, Ridder MC, Montolio M, Capdevila-Nortes X, Polder E, Sirisi S, Duarri A, Schulte U, Fakler B, Nunes V (2011). Mutant GlialCAM causes megalencephalic leukoencephalopathy with subcortical cysts, benign familial macrocephaly, and macrocephaly with retardation and autism. Am J Hum Genet.

[38] Meyer J, Huberth A, Ortega G, Syagailo YV, Jatzke S, Mossner R, Strom TM, Ulzheimer-Teuber I, Stober G, Schmitt A, Lesch KP (2001). A missense mutation in a novel gene encoding a putative cation channel is associated with catatonic schizophrenia in a large pedigree. Mol Psychiatry.

[39] Kaganovich M, Peretz A, Ritsner M, Bening Abu-Shach U, Attali B, Navon R (2004). Is the WKL1 gene associated with schizophrenia?. Am J Med Genet B Neuropsychiatr Genet.

[40] Brignone MS, Lanciotti A, Macioce P, Macchia G, Gaetani M, Aloisi F, Petrucci TC, Ambrosini E (2011). The beta1 subunit of the Na,K-ATPase pump interacts with megalencephalic leucoencephalopathy with subcortical cysts protein 1 (MLC1) in brain astrocytes: new insights into MLC pathogenesis. Hum Mol Genet.

[41] Ridder MC, Boor I, Lodder JC, Postma NL, Capdevila-Nortes X, Duarri A, Brussaard AB, Estevez R, Scheper GC, Mansvelder HD, van der Knaap MS (2011). Megalencephalic leucoencephalopathy with cysts: defect in chloride currents and cell volume regulation. Brain.

[42] Elorza-Vidal X, Sirisi S, Gaitan-Penas H, Perez-Rius C, Alonso-Gardon M, Armand-Ugon M, Lanciotti A, Brignone MS, Prat E, Nunes V (2018). GlialCAM/MLC1 modulates LRRC8/VRAC currents in an indirect manner: implications for megalencephalic leukoencephalopathy. Neurobiol Dis.

[43] Mikhaylova M, Bar J, van Bommel B, Schatzle P, YuanXiang P, Raman R, Hradsky J, Konietzny A, Loktionov EY, Reddy PP (2018). Caldendrin directly couples postsynaptic calcium signals to actin remodeling in dendritic spines. Neuron.

[44] Wales P, Schuberth CE, Aufschnaiter R, Fels J, Garcia-Aguilar I, Janning A, Dlugos CP, Schafer-Herte M, Klingner C, Walte M (2016). Calcium-mediated actin reset (CaAR) mediates acute cell adaptations. Elife.

[45] McCarthy KD, de Vellis J (1980). Preparation of separate astroglial and oligodendroglial cell cultures from rat cerebral tissue. J Cell Biol.

[46] Schildge S, Bohrer C, Beck K, Schachtrup C. Isolation and culture of mouse cortical astrocytes. J Vis Exp. 2013;71:50079. 10.3791/50079 PMC3582677 23380713

[47] Duarri A, Lopez de Heredia M, Capdevila-Nortes X, Ridder MC, Montolio M, Lopez-Hernandez T, Boor I, Lien CF, Hagemann T, Messing A (2011). Knockdown of MLC1 in primary astrocytes causes cell vacuolation: a MLC disease cell model. Neurobiol Dis.

