# Supplementary figures and images for: Plasma membrane localization of MLC1 regulates cellular morphology and motility
Source: Mol Brain. 2019 Dec 30;12:116. doi: 10.1186/s13041-019-0540-6 (PMC6938022; doi:10.1186/s13041-019-0540-6)

Supplementary Figure 1

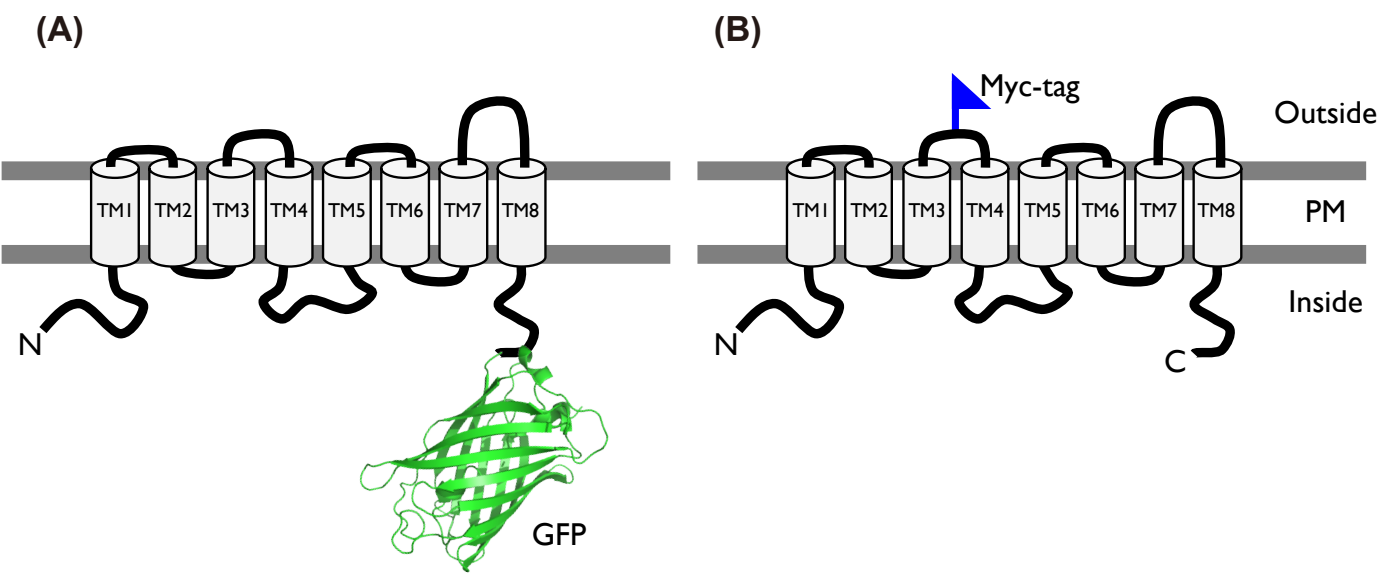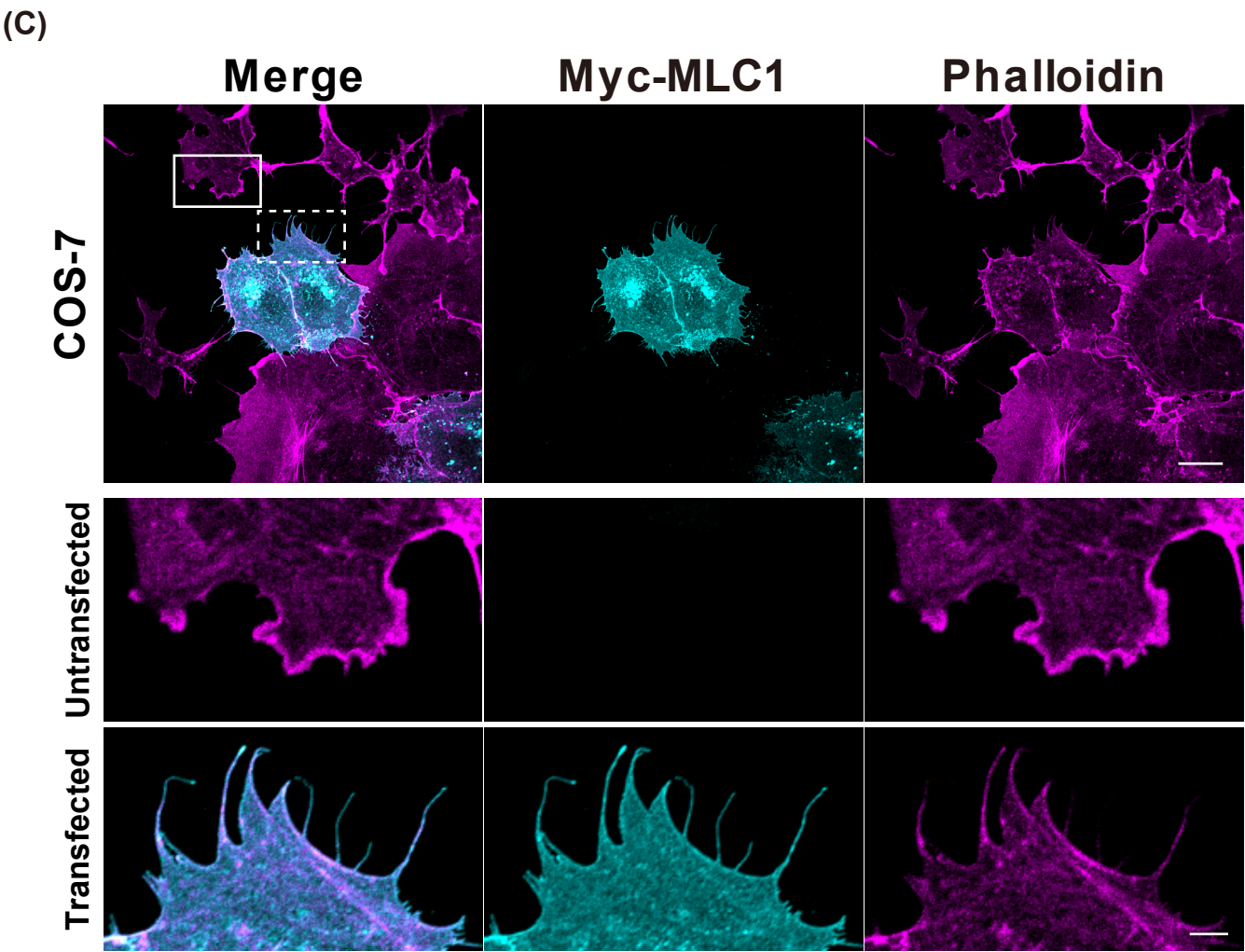

Supplement: Supplementary file 1 — Additional file 1: Figure S1. Myc-tagged MLC1 induces morphological changes. (A) Schematic drawings of a putative transmembrane topology of hMLC1 used in this study. For the hMLC1-GFP construct, GFP was in-framed fused to the 3′-end of hMLC1 (green). (B) For generating Myc-tagged hMLC1, the Myc-tag was placed in the putative extracellular loop of hMLC1 (blue flag). (C) Myc-tagged MLC1 (cyan) also induced the filopodia formation (dotted box) as MLC1-GFP in transfected cell, however, untransfected cell showed lamellipodia (solid box). Fibrous actin was stained with Phalloidin (magenta). Scale bar: 25 μm and 5 μm (magnified image). [file 13041_2019_540_MOESM1_ESM.pdf]

Supplementary Figure 2

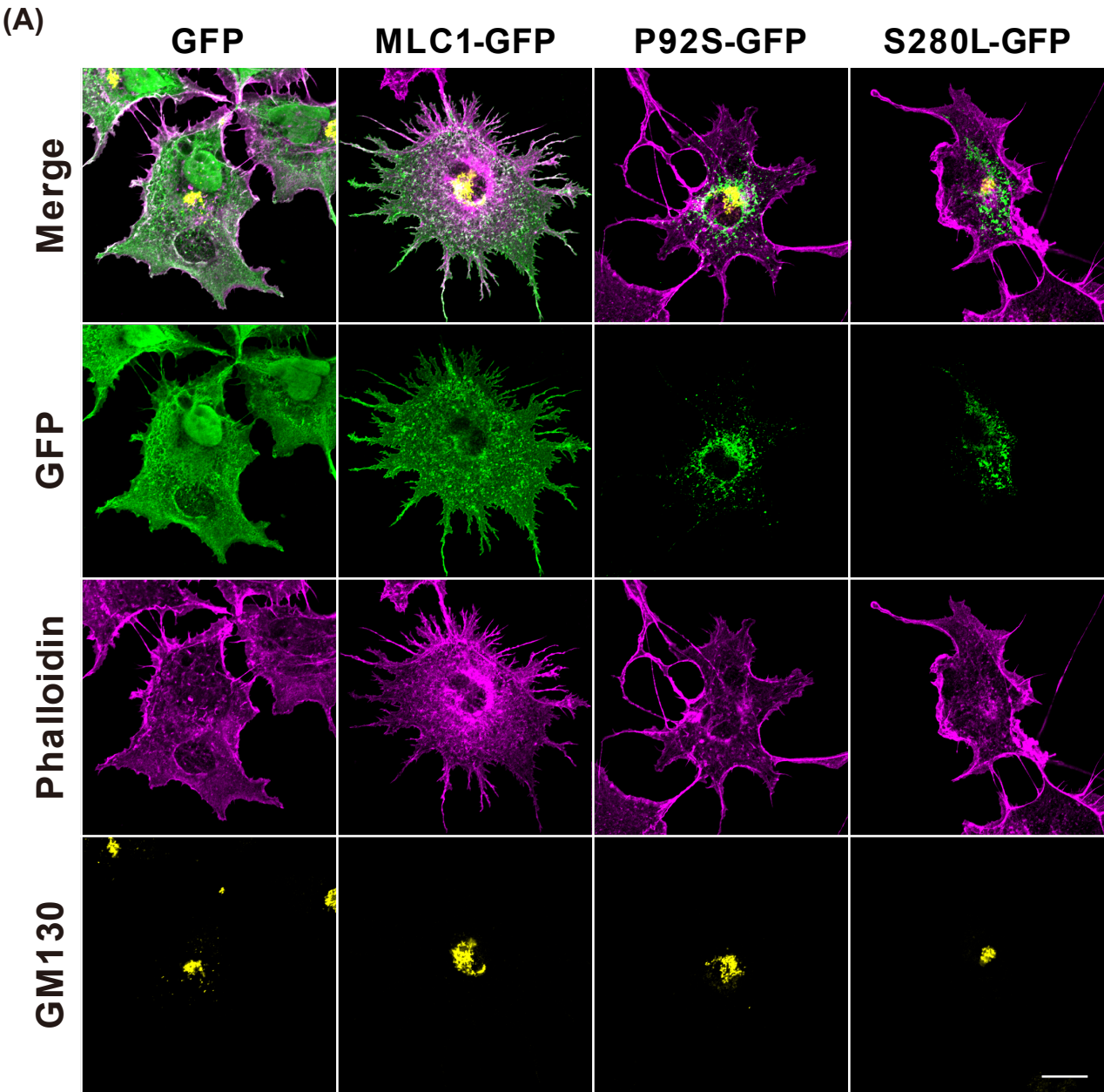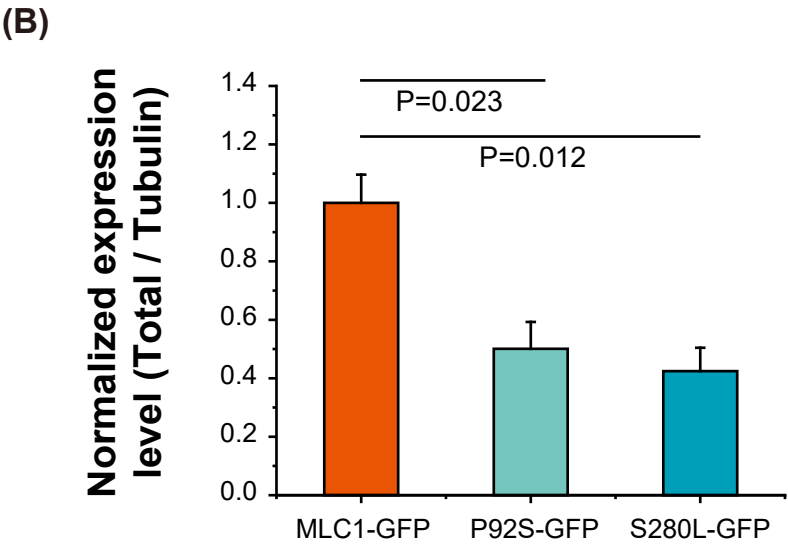

Supplement: Supplementary file 2 — Additional file 2: Figure S2. Partial co-localization of patient-derived mutants and cis-Golgi matrix protein. (A) GFP, MLC1 and patient-derived mutants (P92S- and S280 L-GFP) were expressed in COS-7 cells. Wildtype and mutant MLC1 (green, GFP) were partially co-localized with GM130 (yellow, cis-Golgi matrix protein). Fibrous actin was stained with Phalloidin (magenta). Scale bar: 20 μm. (B) The total expression levels of MLC1, P92S, and S280 L were normalized to the expression level of Tubulin and quantified. [file 13041_2019_540_MOESM2_ESM.pdf]

Supplementary Figure 3

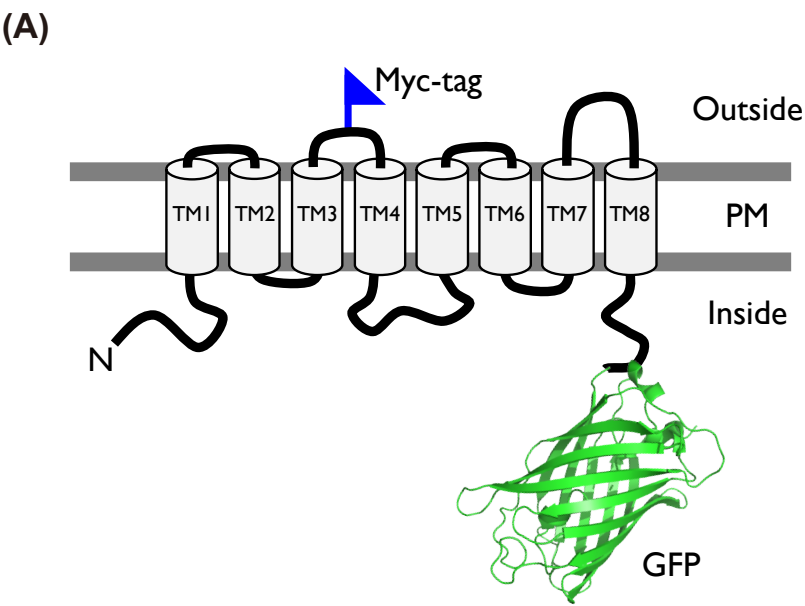

(B)

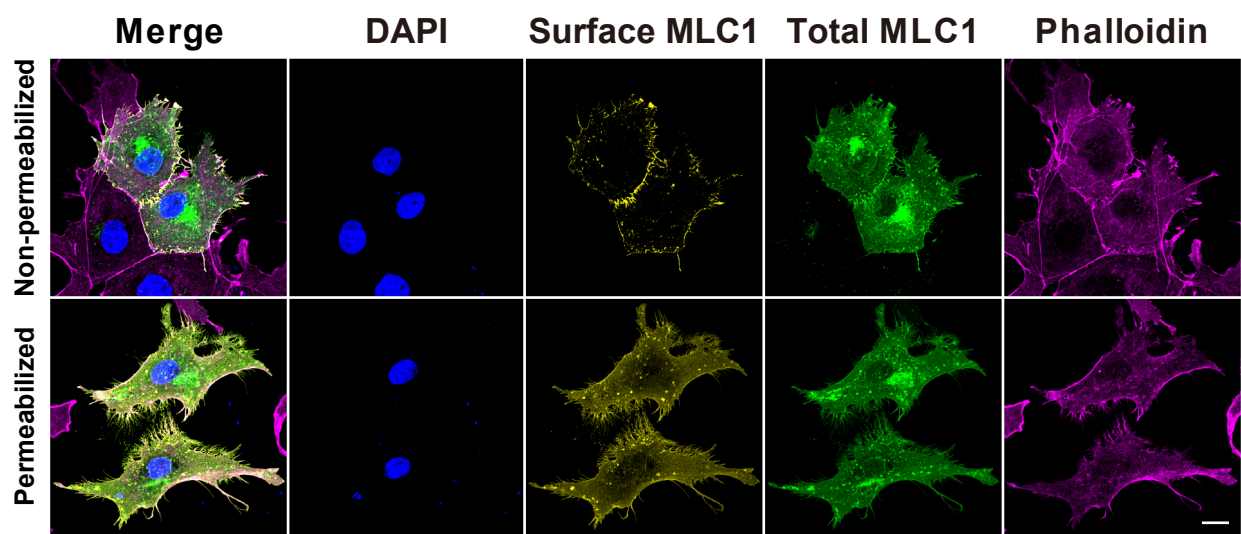

Supplement: Supplementary file 3 — Additional file 3: Figure S3. The antibody accessibility to the Myc-epitope placed in the putative extracellular loop of MLC1. (A) Schematic drawings of a putative transmembrane topology of hMLC1-Myc-GFP. The Myc-tag was placed in the putative extracellular loop of hMLC1 (blue flag, extracellular) and GFP was in-framed fused to the 3′-end of hMLC1 (green, intracellular). (B) For the “Non-permeabilized” condition, Myc epitope in the putative extracellular loop was stained with anti-Myc antibody before permeabilization (yellow, PM-MLC1). After permeabilized, cells were stained with anti-GFP antibody (green, total MLC1), DAPI, and Phalloidin (magenta). For the “Permeabilized” condition, all of staining procedures were performed after permeabilization. Anti-Myc antibody was accessible from both extracellular and intracellular sides only after permeabilization. Scale bar: 25 μm. [file 13041_2019_540_MOESM3_ESM.pdf]

Supplementary Figure 4

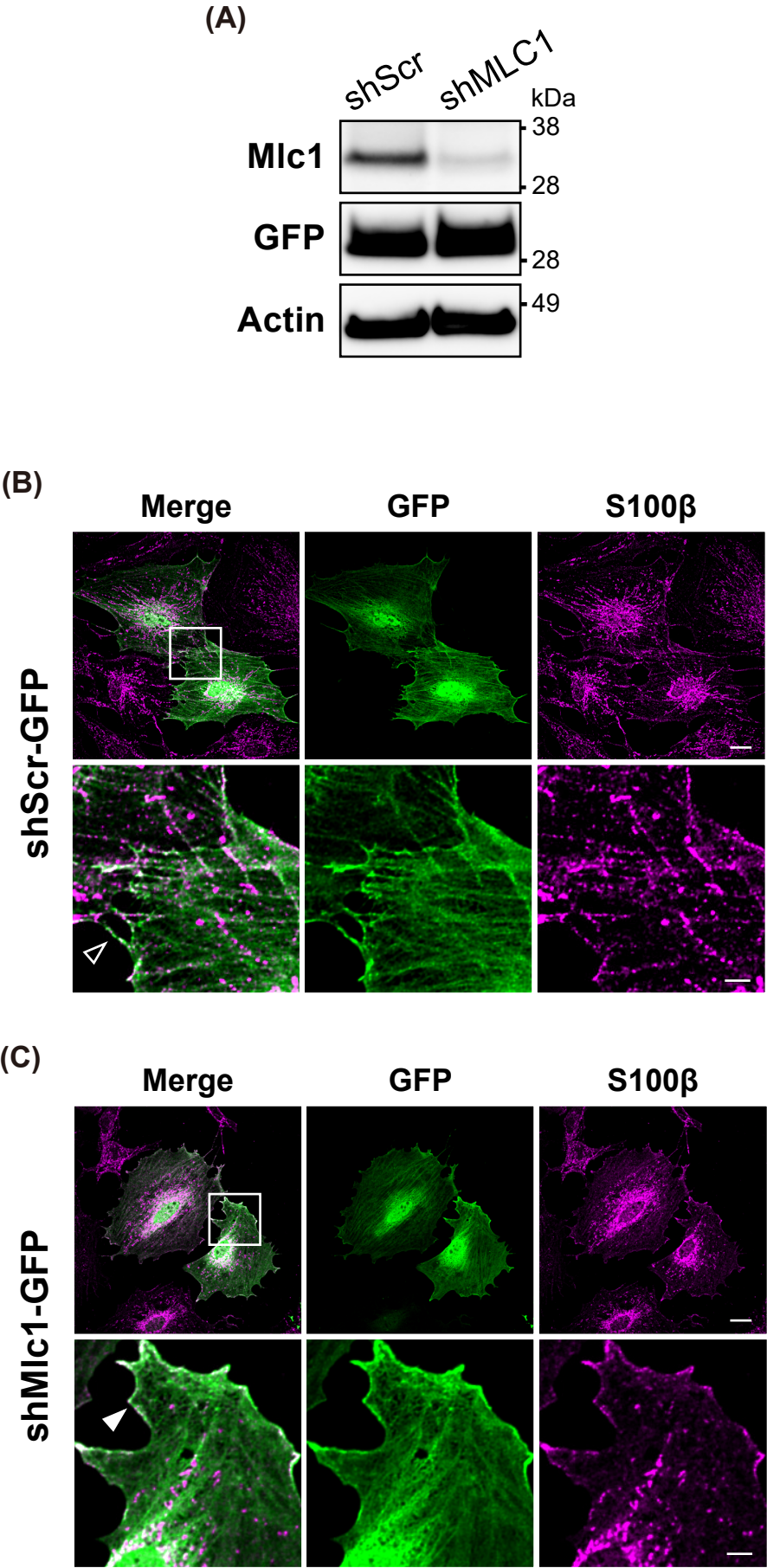

Supplement: Supplementary file 4 — Additional file 4: Figure S4. Examine the infection efficiency and the effect of MLC1 on the matured primary astrocytes. (A) Knockdown of MLC1 in mouse primary astrocytes was performed by viral infection and was confirmed via Western Blotting with anti-MLC1 antibody. GFP expression levels were analyzed to evaluate the efficiency of AAV infection. Similar GFP expression level in the astrocytes treating with either shMlc1 or the scrambled shMlc1 (shScr) virus indicates that the infection efficiency was similar. Actin was used as loading control. (B and C) To verify the maturation of primary astrocytes, cells were stained with anti-S100β antibody (magenta). Empty and filled arrowheads in the magnified image indicate fibrous actin bundles and branched actin networks, respectively. [file 13041_2019_540_MOESM4_ESM.pdf]
